# The Impact of the COVID-19 Pandemic on Those Supported in the Community with Long-Term Mental Health Problems: A Qualitative Analysis of Power, Threat, Meaning and Survival

**DOI:** 10.1007/s10597-021-00932-4

**Published:** 2022-01-15

**Authors:** Dawn Leeming, Mike Lucock, Kagari Shibazaki, Nicki Pilkington, Becky Scott

**Affiliations:** School of Human and Health Sciences, University of Huddersfield, Queensgate, Huddersfield, HD1 3DH UK

**Keywords:** COVID-19, Mental health, Service Users, Power Threat Meaning Framework, Qualitative

## Abstract

Research suggests that the COVID-19 pandemic has had a significant impact on those already living with mental health problems, though there is also evidence of resilience. However, to date there has been limited in-depth qualitative investigation. We interviewed 15 people living with long-term mental health problems who, before the pandemic, were being supported by third sector organisations, to explore how they experienced lockdowns and accessing services remotely. Template analysis was informed by the Power Threat Meaning Framework and suggested that participants experienced significant threats to their mental wellbeing and recovery which were exacerbated by current or previous powerlessness and inequality. Although participants described positive coping strategies, several described a return of unhelpful behaviours that had contributed to the original difficulties. The findings illustrate the wider contributions of social and economic context to mental health problems and the importance of ensuring that people do not feel abandoned and are proactively supported.

## Introduction

In March 2020, the World Health Organisation (WHO) announced the outbreak of the COVID-19 virus had become a pandemic, causing lockdowns and restrictions to be applied across the world. In the UK, three lockdowns were imposed which confined people to their homes for substantial periods of time, preventing interaction with others in almost all social and numerous work settings. This paper discusses the lockdown experiences of a group of people with mental health difficulties who were no longer able to access their usual support from two charitable organisations. Their experiences are explored with reference to the Power Threat Meaning Framework (PTMF, Johnstone et al., 2018), which facilitates a deeper understanding of how mental health difficulties, distress and recovery are shaped by threats to meaningful aspects of life and the negative operation of power.

Prolonged periods of enforced isolation, such as quarantine, have been shown in previous pandemics to have a significant impact on mental health (Brooks et al., 2020). The current pandemic has also posed other challenges that are known to impact mental health, including unemployment, workplace stresses, financial strain, relationship discord, severe and chronic illness and health anxieties (Byrne et al., 2021; Holmes et al., 2020). Therefore, data from 2020 highlighting a significant increase in the number of people experiencing mental health problems, compared to previous years (Daly et al., 2020; Jia et al, 2020), and high levels of stress, anxiety, depression, aggression, lowered self-esteem, alienation, helplessness and insomnia (Abramson, 2020; Shah et al., 2021; Torales et al., 2020), came as no surprise.

Given the reported impact of the pandemic on otherwise mentally healthy individuals, concern has been expressed from the beginning of the pandemic about the potential impact on people with existing mental health problems, many of whom also have poorer physical health and are more exposed to inequalities which may exacerbate the financial and social impacts of the pandemic (Byrne, et al., 2021; Druss, 2020). As Holmes et al. (2020) argued, there is a need for multidisciplinary research to better understand the impact of the pandemic on those living with mental health difficulties, their varied experiences and how services might best respond. To this end, Hao et al. (2020) confirmed that users of psychiatric services living in the community did indeed report more health anxiety, PTSD, stress and insomnia compared to the general population during initial strict lockdown measures in China, though a causal link to the pandemic was unclear. Several studies with people with eating disorders carried out during 2020 also suggested that, for many, lockdown exacerbated problematic eating patterns and feelings of distress (e.g., Clark Bryan et al., 2020; Phillipou et al., 2020; Schlegl et al., 2020). Additionally, a survey of 1434 people living with a range of severe mental health problems carried out during April–May 2020 by the national UK charity ‘Rethink Mental Illness’ (2020) reported that 79% of respondents felt their mental health had worsened due to the pandemic. However, the limited research available suggests that significant difficulties have not been inevitable for those in recovery from mental health problems, certainly in the early months of the pandemic. For example, although 72% of respondents to a USA survey (Costa et al., 2021) felt their pre-existing mental health difficulties had worsened, 50% thought they were ‘coping okay’ and 16% reported that they were ‘coping well’. A longitudinal study in the Netherlands reported that bipolar symptoms increased within the initial phase of the pandemic but then decreased (Koenders et al., 2021), whilst Mergel and Schützwohl (2021) found surprisingly high levels of resilience in Germany amongst those with chronic and acute mental health problems, with difficulties either stabilising or even improving from pre-pandemic to the first few weeks of lockdown, and then to post lockdown social distancing in June–July 2020. Interestingly, a further longitudinal study by Castellini et al. (2020) noted that although a majority of individuals with a pre-existing eating disorder *did* experience worsening anorexic or bulimic eating patterns during the first few weeks of lockdown, this was not the case for all and was more likely for those with a history of childhood trauma and current household discord. Similarly, detailed time series case analyses of daily emotional trajectories for four women with depression and anxiety-related mental health problems demonstrated variation in coping, resilience and emotional response as lockdown restrictions were introduced in Belgium (Dejonckheere et al., 2021). There has been limited in-depth exploration of the particular situations and experiences that may produce these differences, though three qualitative studies with people living with or recovering from mental health problems during the pandemic are particularly helpful—two in the UK (Gillard et al., 2021; Simblett et al., 2021) and one in Australia (Honey et al., 2021). Despite some accounts of coping and resilience, many participants across all three studies had clearly struggled with the pandemic restrictions, describing responses of sadness, fear and anger, negative self-appraisals, worsening mental health and a detrimental impact on wellbeing. However, analyses across the studies suggested that this varied according to factors such as living environment, being a member of a minority group, physical health, social contact, learned coping strategies, sense of control and, importantly, availability of and engagement with mental health service provision. This latter point reflects Costa et al.’s survey finding of an association between poorer coping during the pandemic and difficulties accessing services.

The impact of lockdown measures on services (health/social care, charity, and local community groups) has resulted in fewer face-to-face contacts, and people with mental health problems appear to have been disproportionally affected by this (Holmes et al., 2020; Orhan et al., 2021; Weissman et al., 2020). Many healthcare staff adapted their usual working practices and sought new ways to support their clients, for example by remote working and telephone support. However, although some clients found telehealth support helpful (Honey et al., 2021; Venville et al., 2021), this has not been successful in all situations, with concerns about remote relationship building and inequality of access to technology (Liberati et al., 2021), and many healthcare staff expressing concerns about the deteriorating mental health of their service users (Johnson et al., 2021).

Despite a significant number of studies focussing on the link between the pandemic and the mental health of the general population, there is still limited research with people living with long-term mental health difficulties, and how they experienced and coped with the lockdown and with accessing mental health services remotely (Gillard et al., 2021). There is even less research available yet for this group on the cumulative effects of repeated periods of restrictions. Moreover, the limited research has tended to focus on reduced support and provision from mainstream mental health services, with less attention to changes in support offered by community and third sector (charity) organisations. As noted by Honey et al. (2021), there is limited understanding of the impact of restrictions in psychosocial support services. Services offered across the third sector include talking therapies, crisis support, peer support, creative arts, advocacy, welfare benefits advance and complementary or alternative therapies. They are recognised as important in supporting ongoing recovery from mental health problems and are increasingly linked to social prescribing schemes (Bickerdike et al., 2017). These organisations, whether large national charities or small independent charities serving a local community, are often founded on different perspectives to mainstream mental health services. They may require different roles for supporters (e.g., Longden et al., 2017; Naylor et al., 2013), with volunteers and people with lived experience of mental health problems often working alongside paid staff. Many community-based services emphasise normalisation, empowerment and supporting personal meaning in life (Borg & Davidson, 2008), and provide flexible psychosocial support for longer term recovery. Therefore, they are likely to play a different role in the lives of service users than mainstream clinical services.

The current study focused on exploring the experience and challenges of pandemic restrictions for people living with long-term mental health problems who, prior to the pandemic, had accessed support from two community-based third sector providers of psychosocial support that was available to all without payment. Particular attention was paid to the impact of the pandemic on recovery and to how service users managed their mental health difficulties. The findings presented below are part of a larger project which included practical consideration of which aspects of remote services had worked well and what services might be able to do differently. Here, we offer an analysis of the participants’ experiences that draws on the Power, Threat, Meaning Framework (PTMF, Johnstone et al., 2018) in order to understand how the pandemic context shaped or impeded recovery and how participants responded to this. The PTMF argues that the thoughts, feelings and behaviours experienced during worsening mental health can often be understandable as meaningful survival responses to threats that have arisen through the negative operation of power. The operation of power, and consequent experience of powerlessness, is considered central to the development of mental health difficulties, with power conceptualised as taking many forms related to wider economic, social and legal structures, as well as more immediate forms of interpersonal, embodied and coercive power. The PTMF draws on extensive research demonstrating the impact on mental health of disempowerment in various forms such as bullying, abuse, poverty, unemployment, violence and various forms of discrimination and inequality. The focus then is on what has happened to disempower and threaten people rather than what is ‘wrong’ with them. As such, the framework is presented as an alternative to the medical model of mental illness, rejecting the idea that commonalities in emotional and behavioural responses to threat and disempowerment should be viewed as ‘symptoms’ of ‘disorders’ but instead as understandable survival responses. Aherne and Aherne (2020) note that the PTMF may offer a helpful perspective for understanding psychological distress related to the COVID-19 pandemic. In most countries there have been periods of significant legal constraint on the behaviour of the population, with particular threats to health and wellbeing, personal freedom, livelihoods and connections with others that may have exacerbated prior social inequalities and feelings of powerlessness. Therefore, our analysis focuses particularly on the threats that the participants had experienced due to the pandemic, how these related to prior inequalities and experiences of disempowerment, the sense they made of these threats, and the strategies participants used to cope.

## Methods

### Overview

A qualitative design was used with semi-structured individual interviews focusing on experiences of lockdown and periods of reduced social contact.

### Setting

The research was carried out in a local authority area in the North of England with a population of 210,000, 90% of whom are White, and 8.5% Asian/Asian British. It has an Index of Multiple Deprivation (IMD) of 89 out of 317 local authorities in England (1 is the most deprived). Participants were recruited via two charitable organisations. Organisation A aims to increase the health and well-being of local communities, with an emphasis on those living with mental health issues who are isolated and lonely. Before COVID-19 the organisation offered a number of group activities, using music, art, writing, beauty therapy and exercise to reduce isolation and build confidence and self-esteem. During lockdown, face-to-face support was suspended though some telephone support, and limited online support, continued. Eight interviews with users of Organisation A were conducted during phase 1 in September–October 2020. This was after the first full UK lockdown and the re-establishment of limited face-to-face group activity. However, rates of COVID-19 were rising again, and restrictions had been re-introduced in the local area, including closure of hospitality venues such as restaurants and bars, prior to a second national full lockdown in November–December. Organisation B is a user-led organisation run by and for disabled people. It seeks to empower and enable disabled people with physical and/or mental health issues, by providing them with information and support. Before the start of the pandemic, Organisation B held weekly drop-in meetings. Interviews with seven users of Organisation B were conducted during the third national UK lockdown in January–February 2021 (phase 2) while drop-in meetings were again suspended.

### Participants

Participants were required to identify as having, or being in recovery from, mental health difficulties and to have received support from one of the community organisations. Invitations were not sent to anyone currently in crisis or unable to give informed consent. The 15 participants were between 45 and 65 years, with an average age of 54 years. Eleven were women, four were men and all were of White British nationality. Formal diagnoses were not recorded, but participants referred to a range of difficulties including psychotic symptoms, depression, anxiety, eating disorders, bipolar disorder, personality disorder and trauma related to domestic violence and abuse during childhood. All had experienced mental health problems for at least 2 years, with most experiencing difficulties for several years. All lived in their own homes, though four were either in housing that provided additional support or had received visits from carers prior to the pandemic. Seven had also been receiving support from either mental health services (National Health Service—NHS) or a therapist, and two of these had recently been hospitalised. Three received mental health support only from their general practitioner or Mind (national mental health charity) and five had not been receiving any additional mental health support immediately prior to the pandemic, although two of these were on waiting lists for therapy or NHS mental health services. Eight disclosed physical health problems or disabilities including use of a wheelchair, type one diabetes, Myalgic Encephalomyelitis/chronic fatigue, forthcoming surgery and osteoarthritis. None had been in full-time paid employment or full-time education prior to the pandemic, though several had undertaken voluntary work.

### Recruitment and Consent Procedures

Recruitment was carried out via the community organisations who sent out information sheets to people likely to meet the study inclusion/exclusion criteria. Those who indicated interest in taking part were contacted to check eligibility, take consent and arrange a time for the interview. Consent was rechecked at the start of the interview and participants were informed that any identifying information would be removed to ensure anonymity. All were given pseudonyms.

### Interviews

Semi-structured interviews were conducted by telephone due to restrictions on face-to-face contact and limited access to other technology for many service users. A topic guide was designed following discussion with staff working in Organisation A about the concerns of their clients. This covered participants’ current living situation, their contact with others, their experiences of and feelings about the previous or current lockdowns and suspension of face-to-face support services (positive and negative), the impact of this on their mental health, their support experiences during the pandemic, strategies they had adopted for coping and ideas about ideal future support. Framing of questions was informed by the PTMF (Johnstone et al., 2018), focusing on what had happened to participants during lockdown, how this had affected them, the meaning of this and what they did to cope. Participants were not asked directly about more abstract concepts such as threat and power, but interviewers probed sense of control and the challenges they had faced. Interviews lasted between 30 and 150 min (with a break), were audio recorded and transcribed verbatim.

### Approach to analysis

Template Analysis was used, a form of thematic analysis involving hierarchical coding through the iterative development of a coding template (King & Brooks, 2017). It sometimes involves the use of a priori codes to draw attention to areas of theoretical interest but can be entirely inductive. To check that participants’ concerns were not overshadowed by use of the PTMF, initial coding and clustering of codes was carried out inductively by BS and NP on six interviews. The research team then reflected on the emerging themes, discussed their relevance to the PTMF and agreed the framework was a good fit. A template was then constructed in an NVIVO database, with the following higher order themes developed to incorporate and expand the initial inductive codes, but interpreted with attention to power, threat and meaning: (i) Threats to Participants from Pandemic, (ii) Meanings of Threat and Pandemic for participants, (iii) Prior Inequalities that Exacerbate Threat and (iv) Responses to Threat. These higher order themes were then used by NP and BS for re-coding the original six transcripts and coding the remaining transcripts. Further interpretative analysis by the team led to adjustment of overarching theme titles and merging of the first two themes, as presented below.

### Compliance with Ethical Standards

The study was approved by the School of Human and Health Sciences Research Ethics and Integrity Committee at the University of Huddersfield and all participants provided informed consent for both participation and publication. The authors have no known conflicts of interest, and all certify responsibility for the manuscript.

## Findings

**Fig. 1:**
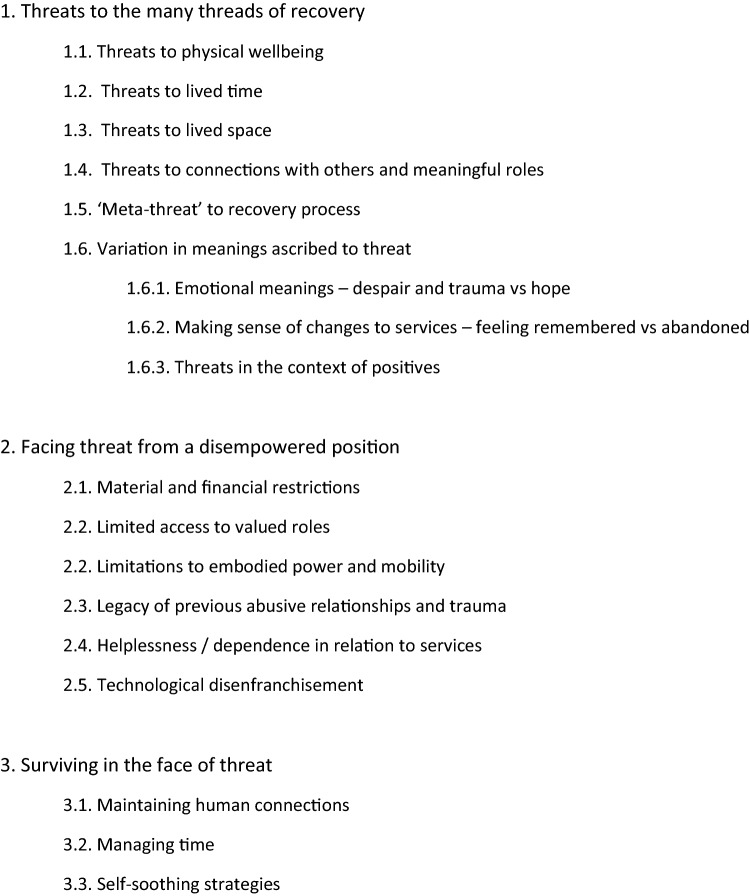
Themes and sub-themes

Analysis suggested overall that participants had experienced significant threats to their mental wellbeing and recovery due to the pandemic and related restrictions. These threats were exacerbated by situations of current or previous powerlessness and inequality. However, many described drawing on past experience to manage mental wellbeing actively, though sometimes self-soothing involved a return of behaviours that had contributed to the original difficulties. These findings are discussed in more detail below under three themes (see Fig. 1 for a list of themes and sub-themes).

### Theme 1: Threats to the Many Threads of Recovery

During lockdown, participants experienced threats to many aspects of their day-to-day experience which could pose significant challenges to recovery. We were struck by the correspondence of our initial coding of threats to van Manen’s (1997) four lifeworld existentials which he proposed as capturing all dimensions of lived experience: lived body, lived time, lived space and lived human relations. Participants experienced destabilising threats across all of these, with threats to one dimension exacerbating threats to another. Those who disclosed physical health problems or disabilities were the most concerned about threats to physical wellbeing such as catching COVID-19, breathing difficulties, and reduced access to physical health services or food. However, across the group as a whole, there was a greater focus on threats to structure, routines, movement, activities and relationships, with these sometimes exacerbating threats to physical wellbeing. Therefore, aspects of life which have previously been identified as foundations for recovery from mental health problems (e.g., connection, meaningful roles, empowerment, Leamy et al., 2011) became threatened and uncertain.

Disruption to the experience of time was troubling for many as they lost stabilising routines and meaningful, enjoyable occupation. Several participants talked about an altered perception of time as dragging or stretching indefinitely. Jane (P1^1^) explained the impact of losing her routine:

*…Its kept me sort of like a regimental sort of thing, routine. It’s just a routine. If I don’t that, that’s what sends me crazy….. sends me unbalanced.*

Loss of freedom and control over space was also experienced as destabilising, including loss of green spaces, preferred social spaces or access to ‘safe’ spaces that were seen to ‘*really help your mental health*’ (Cathy, P2). Some responses also illustrated the way these restrictions could exacerbate previous difficulties:

*… that’s made my fear worse, because I have to build myself up to go out anyway* (Liz, P2).

*I don’t have much social skills anyway, err, so obviously they’re going to decline* (Dave, P1).

For several, prior to the pandemic, the support organisations had provided not just opportunities to practise social skills but an important sense of belonging with people with shared experiences, that they did not necessarily experience elsewhere—‘*You feel wanted*’ (Naomi, P1). It was a ‘*lifeline for a lot of people, …they’re still part, … of being and doing*’ (Amy, P1). This sense of belonging to the community of Organisation A was now threatened and loss of what felt ‘human’ about contact was mourned, even when using technology such as Zoom to meet online. A few had ‘*learnt to reluctantly embrace Zoom*’ (Ian, P1) for some tasks. However, although a couple of participants mentioned the benefits of receiving more phone calls from services, remote support was not sufficient for many people. Anxiety about unfamiliar technology or the absence of face-to-face contact prevented them feeling fully present in a group and vital support from voluntary and other services was no longer experienced in the same way:

*So, I have been able to talk [online & via phone] to people from the [support] group which is good… But, it’s just, not seeing them no more …That’s gone and everything, since the lockdown* (Naomi, P1).

*I need to see you face to face [counsellor] because I don’t always understand things, I can’t deal with my feelings on my own…So, I’m on hold* (Liz, P2).

*I speak to them [on phone] and I’m happy to hear from them, but it just reminds me of what I’m missing* (Jane, P1).

Restrictions on social contact also threatened the ability to support others. Some expressed worry, helplessness and frustration about lockdown restrictions preventing them caring for family members, whilst others found that meaningful roles they had developed as part of their recovery were no longer available to them:

*Well, I used to go to [Organisation A] three days a week and I used to go to [Redacted] one day a week … and support people with various issues ... I had connections to things from before, like attending high level staff meetings for [Redacted]. I went to do things with [Redacted], so all of that’s gone* (Mark, P1).

Many of the accounts suggested that the above threats posed a ‘meta-threat’ to the recovery process, and some participants made this explicit—“*it’s probably pushed me back two years*” (Ian, P1), “*I feel like I’ve gone back down to rock bottom again*” (Liz, P2). Some participants talked as if the scaffold for their recovery had gone, whether this was the loss of routine, safe spaces or connections with others. Lucy (P2) expressed this particularly eloquently:

*… normally I've got a really good tool bag to manage myself, and what I've found since the Covid stuff is that… Okay, so normally you would, even if your internal world was going to shit a little bit, you could put your anchors out to the outside world and those threads would pull you back in. But it seems like there isn’t anything on the outside to stabilise yourself, so your internal world might be going mental, but so is the external world, … because everything’s subject to change …. nothing’s solid.*

Others referred to increased problems such as addiction or eating, panic attacks, worsening depression, increased voice hearing and, paradoxically, Ian (P1) noted how the respite from the demands of social interaction was ‘*not really helping to address my initial problem which was … getting out and interacting with other people*’*.* There seemed an assumption across many interviews that the negative impact of lockdown on mental health was self-evident. Most talked of their mental health difficulties as something that “*normally I’m able to self-manage*” (Keith, P2), to some degree. However, this had been challenged by the pandemic.

However, as suggested by the PTMF, not all challenges and losses were experienced as similarly threatening by participants. The meanings ascribed to them varied considerably. For some, particularly when restrictions had continued for several months, the situation had strong emotionally ladened meanings with a generalised sense of threat, loss of confidence, shock, trauma, and despair about a situation they felt they could no longer cope with. Even by the first phase of the study, Jane’s interpretation of the situation as ‘never ending’ made it difficult to bear:

*… I’ve got more frustrated as the weeks have gone on. I’ve been coping and coping and focusing….. now lately, it’s just got too much for me. Just now it’s just hit me and I just think because it’s never ending and now I’ve realised that the time has gone on so much, I can’t do it no more and I felt like I couldn’t take it no more. I felt very, very low.*

For others, the emotional impact seemed less, with a stronger sense of hope for normality returning, and recognition of the positives of improved community relations, despite their struggles. Loss of or interruption in services could be experienced very differently depending on whether there was a perception of being abandoned or a sense that services cared but were struggling. Communication from services was key to shaping participants’ meaning-making about threats to their connections with support. Several mentioned how they valued services checking in on them proactively by phone and text and other concrete indicators that individuals were remembered rather than abandoned:

*‘it were extra special that, a phone call, you know, when you’re on your own’* (Dave, P1).

*I’ve had [worker from Organisation A] ringing me up, asking me if I’m alright, you know…. And then, we got a parcel sent to everyone at [Organisation A], with a jig-saw in, quizbook, workbook, a cup, a card to say that they’re missing us. You know, it’s so nice* (Naomi, P1).

Where this was missing, it was noted:

*Bit annoyed really, I thought (X team) would keep in touch a bit more. I’ve rang them two or three times but that’s it…nothing…it would have been nice if somebody… could have rang me* (Lynda, P1).

Participants were asked if there were any positive consequences of lockdown. Some mentioned respite from the usual demands of life, personal achievements, increased self-understanding, and societal changes such as environmental awareness and community cohesion, though these were less often mentioned spontaneously. However, there was a much stronger emphasis on how the pandemic had threatened their wellbeing and recovery.

### Theme 2: Facing Threat from a Disempowered Position

The PTMF suggests that threat arises from the negative operation of power. Many of the threats experienced by the participants were a consequence of new powers operating throughout UK society such as legal restrictions that affected the population as a whole. However, for a number of the participants, prior inequalities and abuses of power, and their dependence on services exacerbated the impact of these threats and their ability to mitigate their situation. Loss of valued social roles, such as volunteering, had greater impact where alternative roles were not readily available (see Mark’s comment above). As Kate (P1) noted: “*it can be so much worse in lockdown for people that are already in a shit situation.*”

Restrictions on use of space were harder to cope with for those who were materially disadvantaged, on low incomes, in poor housing or with restricted mobility. Several had also recently experienced flooding. Cathy (P2), who used a wheelchair and had been trying to move flat for 18 months, explained that despite her extreme fear of catching severe COVID, she had broken several months of indoor exile when she could no longer cope:

*My upstairs neighbour … was jumping up and down and playing loud music and it just got to the point where I just thought I can’t take this anymore.*

Mary (P2) compared struggling in ‘*unsuitable*’ housing without welfare benefits during the first lockdown, to her current situation where her new house gave her stability, quiet, and access to a park—‘*it saved my life*’*.*

Five of the women alluded to the legacy of prior abusive relationships and trauma which could exacerbate strain, add to anxiety around following rules and the risk of sanctions, or make fraught lockdown relationships harder to handle. As Clare (P2) said, ‘*I just go into a horrible little whirlwind*’*.* Lucy (P2) explained that higher levels of current stress meant “*things trigger me, much, much quicker than they generally did before*”*.* Liz (P2) added:

*We’ve had to deal with this [pandemic], as well as what we’re already dealing with from our past with whatever’s gone on. So, this is double impact, you know. So, it takes you right down.*

Many spoke about their relationship with currently suspended services as something they had little control over. Being on a waiting list or ringing again and again could heighten anxiety and a sense of helplessness and dependence, particularly where people could not access support with disability related issues, health problems or financial benefits. Several noted their lack of access to or discomfort around IT—*‘I find the, erm, you know, the internet and the technology just too much*’ (Tina, P2). This disempowered people by excluding them from both online interaction and the dominant method of accessing benefits and advice.

### Theme 3: Surviving in the Face of Threat

Despite the struggle many had experienced, and acknowledgement of their limited control in certain aspects of life, many described taking active steps to make the best of their situation, drawing on their experience of recovery over a number of years. Several responded at length to questions about coping and described strategies that mostly fell into three main areas: maintaining human connections, managing time and self-soothing strategies.

#### Maintaining Human Connections

This was important to some extent for all participants. Ian (P1), as well as others, noted that during lockdown he had ‘*talked much more, to my neighbours*’. Some reflected on a shift in perspective to greater valuing of community. Several mentioned the UK weekly ‘Clap for Carers’:

*The community effect of everybody clapping and applauding together from their own separate homes was incredibly important and very, very uplifting* (Cathy, P2).

Between the full lockdowns, some reached out to other users of the support organisations, particularly when they were struggling. Access to and familiarity with technology varied, but several participants maintained connections with others remotely. Cathy (P2), who was shielded due to physical health problems, said that continuing her voluntary telephone role ‘*made me feel really useful*’*.* One organisation had provided tablet computers to maintain contact which were appreciated by some, as was the continuation of some online classes. However, participants adopted a cautious and pragmatic approach, weighing the value of different forms of online connection. Kate’s (P1) summary captured several responses:

*….. Those little drops of support are so hard to find nowadays that you just take what you can wherever. Although I don’t think support is good over Zoom, classes are good over Zoom and that can be part of your self-care, which I think is really important…..just to try and build that resilience any way you can really’.*

Ironically, the same organisation had posted the much cheaper parcel with card mentioned above. This low-cost gesture seemed to provide a stronger sense to some participants of feeling supported and connected than could be achieved using unfamiliar connective technology.

#### Managing Time

Maintaining and adapting routines or constructing new routines and keeping occupied seemed an important means of gaining a greater sense of control. Keith (P2), along with others, noted ‘*I structure my day a bit more now*’. Lucy (P2) noted how this came to the fore at certain times:

*Well, sometimes you're alright with it, aren’t you, because you're just like, sod it… let’s just go with it. Then other times you get this extreme need for routine and structure.*

Several were able to draw on established hobbies such as sewing—‘*it’s definitely my therapy blanket*’ (Kate, P1), or developed new hobbies and skills, all of which could give a sense of pride and achievement, as Lynda (P1) indicated:

*I’ve had a lot of successes. You should see the size of my cauliflowers (laughs)….it makes a big difference.*

Other participants mentioned the importance of regular scheduled contacts with others, whether face-to-face or via phone. Even if there were doubts about online meetings/classes as a means of connecting with others, they helped to manage time. As Lynda (P1) noted, ‘*it takes your mind off things for a while*’.

#### Self-soothing Strategies

Although a number of participants talked about drawing on longstanding strategies which helped their resilience, several noted that they were managing their moods and self-soothing in a way that could undermine recovery. One disclosed resuming amphetamine use during lockdown to manage boredom. She and three others also noted increased alcohol use and three with previous eating disorders said they had returned to restricting their eating or exercising excessively. Jane (P1) attributed her ability to overcome her problems with restrictive eating and exercise to her attendance at a support group prior to the pandemic. Being isolated again ‘*made me very ill*’. She returned to restrictive eating and exercise as a way of coping:

*The only problem is I’ve found these ways of coping, but on the other hand, it’s like the eating thing and the exercise thing, I love it though, but it took over again … my mum said you’re going to collapse because you’re walking too much…and I still go out whether I’m in pain or not….and I’m worn out.*

Therefore, recovery was not just derailed during lockdown by threats to services, relationships, physical wellbeing and control over time and space, but also by the strategies some participants used to cope with these threats. However, some talked of using journal writing, positive thinking, creativity, CBT techniques, breath work, lavender oil, crystals, maintaining soothing home environments, meditational music, time outdoors and even singing in the rain. Lucy (P2) spoke of incorporating positive thinking into her day:

*..You have to have a gratitude practice, that’s one of the things that I do to keep myself together. I wake up every morning, I think, okay, I'm still alive, I've got a roof over my head, things might be tricky, but we've got enough food. I've got my family around me…*

Many of the participants were able to draw on years of experience managing their mental health. They did not take good mental health for granted: ‘*I’ve got a very large resource toolbox which I’ve been building on my whole life*’ (Cathy, P2). However, the pandemic had stretched their coping skills considerably, particularly those who were multiply impacted by isolation, prior trauma, poor accommodation, caring responsibilities and financial concerns.

## Discussion

This study highlights the various impacts of the COVID-19 lockdown and restrictions on people living in the community with mental health problems. The qualitative methodology has allowed an in-depth understanding of the challenges to their recovery and provides revealing insights into what is important in long term recovery and effective management of ongoing mental health problems, particularly for people living in difficult life circumstances. The lockdowns clearly had wide ranging effects and threatened the ways in which participants were managing their mental health problems and trying to stay well, which applied across all four aspects of the lifeworld (van Manen, 1997)—the body (physical health), lived time (routines and activities giving structure to the day), space (restrictions on places where people can go to engage in meaningful and beneficial activities), and human relations (face-to-face social contact). All this was in the context of disempowerment and a loss of control over these key aspects. Leamy et al (2011) proposed the empirically based CHIME framework incorporating five key recovery processes—connectedness, hope, identity, meaning and empowerment, and Stuart et al. (2017) suggests a sixth process, ‘difficulties’, which acknowledges the struggles that people face in recovery. This study supports the importance of some of these processes such as connectedness and empowerment, regardless of the pandemic situation, but also highlights the challenges that particular difficulties, such as lockdown restrictions, pose for sustained recovery. Therefore, much that has been considered important for recovery was still important during this period. However, what seemed additionally troubling was the removal of the recovery scaffold that participants had put in place, in terms of routines, structure and relationships.

Much of the research on the impact of COVID on those with mental health difficulties has been concerned with understanding whether difficulties have worsened due to the pandemic and whether recovery is more threatened and relapse more likely. Although our participants talked about losing parts of their recovery scaffold, it is difficult to know to what extent this increased the likelihood of a relapse. Relapse rates are difficult to measure, with definitions and measures varying across studies. Researchers have often relied on rates of hospitalization as a proxy for relapse rates (Addington et al., 2013), which are problematic to interpret within a pandemic when many health services were discouraging inpatient admissions. Moreover, some mental health problems, such as depression, can be experienced as either a long term or re-occurring condition (Solomon et al, 2000), so reducing relapse rates and supporting self-management, rather than complete absence of relapse, would be positive outcomes. The limited longitudinal research, reviewed in the introduction, indicates a mixed picture with regard to whether mental health difficulties have generally worsened. However, the above understanding of recovery and the PTMF would suggest that we need to look beyond relapse rates and measures of symptomatology to understand whether and how the ability to recover in the sense of living a meaningful life and feeling a sense of wellbeing might have been impeded. To our knowledge, there has not been any detailed longitudinal qualitative research providing insight into changing experiences of recovery in a broader sense as the pandemic unfolded. However, in the present study, for some participants, the setbacks associated with the pandemic were certainly seen as ‘undoing’ previous recovery and re-traumatising people, leading to concerns that they would not be able to get back to where they were before the lockdowns. It was also apparent that threats and setbacks are much harder to deal with if a person is experiencing broader social inequalities and multiple health problems and if there is a sense of having been abandoned by the usual sources of support. As Castellini et al. (2020) noted for people with eating disorders, the present findings also suggest that earlier trauma may have made it more difficult to tolerate aspects of lockdown. This is a reminder that understanding the impact of the pandemic on those with mental health difficulties needs careful attention to context. As suggested by Dejonckheere et al. (2021), our findings indicate that for some people with pre-existing mental health difficulties in some circumstances, the pandemic has had a profoundly negative impact on recovery, though the impact for others may have been less. Our findings also suggest that adverse consequences of lockdown may have become greater over time for some individuals. Although it is difficult to be certain of this without a longitudinal design, the accounts of some participants in the second phase suggest that studies focusing on the early weeks of the pandemic may not capture the scope of later distress.

The PTMF was a useful way of piecing together the findings of this study and the findings also provided some support for the framework. For example, it was clear that the pandemic presented various threats which were experienced differently by different people, that prior inequalities and experience of disempowerment exacerbated threats and restricted options for coping, and that responses to the threats were understandable but sometimes helpful and sometimes not. Not surprisingly, and in line with recent surveys (e.g., Khosravani et al., 2021; Phillipou et al., 2020; Rethink Mental Illness, 2020; Schlegl et al., 2020), some participants reported a return to coping strategies which could undermine recovery such as use of drugs and alcohol, compulsive behaviour, excessive exercise and restricted or excessive eating. Using the PTMF demonstrates the limitations of over-generalised and passive notions of individual vulnerability to stress noted by Johnstone (2000). Our analysis suggests the importance of more individualised, complex and contextualised understandings of how adversity impacts people differently, depending on the meanings ascribed and the actions individuals take in response, within the limits of the choices available to them.

There are several implications arising from this study. The first is the importance of acknowledging the wider contributions of social and economic context to mental health problems, not just attributing it to personal vulnerability. These include psychosocial stresses, such as those arising from the pandemic, wider social inequalities which restrict a person’s autonomy and options for coping, and childhood adversity which can also disempower by impacting current coping (Boyle, 2020). The importance of face-to-face personal support and the limitations of remote support were also apparent. It is possible that whilst psychoeducational interventions may be acceptable and helpful when delivered remotely, emotional support may be better provided face-to-face when possible. This is contrary to research suggesting no significant interactional differences between telephone and face-to-face psychological therapy (Irvine et al., 2020). Echoing others (Holmes et al., 2020; Liberati et al., 2021), we suggest more research is needed to understand what works best for whom in different contexts. It was also striking how participants differed in the meaning they attached to a reduction in support, and how relatively low-cost interventions such as regular but brief contact to check how people were and the ‘well-being hamper’ helped participants feel supported and not abandoned. The perception that people felt remembered and cared about seemed to help them to cope with the reduction in support available. It also seemed to become harder to stay well, retain hope and plan ahead as the pandemic continued and further lockdowns occurred so it is important for services to bear this in mind and continue to provide support and hope that people will be able to get ‘back on track’ in their recovery journey. Finally, as found in other studies (e.g. Dejonckheere et al., 2021; Simblett et al., 2021), many participants were remarkably resilient and resourceful, drawing on their experience of managing pre-existing difficulties, despite the new threats posed by the pandemic. As service users have noted elsewhere (Gillard et al., 2021), there may be limits on the sustainability of some self-management strategies without access to formal support. However, whilst not overstating the possibility of personal resilience, it is important to reinforce it and help people to recognise it in themselves (Dejonckheere et al., 2021), and in how they can support others during a period of shared adversity. It will be useful for all services, not just clinically-orientated services, to provide opportunities for service users to reflect on and make sense of the adversity they faced during the pandemic and their ways of coping with it. Being able to frame returning ‘symptoms’ as understandable responses to threatening circumstances, that may be shared by others, can be therapeutic. It helps avoid stigmatising assumptions that people are ‘abnormal’, ‘disordered’ or to blame for their problems which can have a significant impact on recovery (SHIFT Recovery Community, 2020). Providing alternative narratives of distress is in itself empowering (Boyle, 2020).

There are a number of limitations for this study. The sample was restricted to one local authority area in the North of England. Due to the difficult circumstances in which services were working it proved difficult to engage other organisations. Therefore, the sample was relatively small and not as diverse as we would have liked, and the findings cannot be taken as capturing the full range of pandemic experiences for those with long-term mental health difficulties. Transferability of the findings will be greater in situations with some similarity to the situations of the present participants. Using telephone interviews enabled inclusion of those with limited access to technology but may also have hindered engagement for a few participants where interviews were shorter. There are likely to have been limits within a single research interview to the detail some participants felt comfortable disclosing about the meaning of threats, their personal history and their responses to challenging circumstances, and the format may not have facilitated full awareness of more distal sources of power and threat which can be difficult to identify (Boyle, 2020). The possibility of repeated lockdowns was not predicted at the start of the project and therefore the initial participants were recruited for single interviews. This meant that it was not possible to track changes in the experiences of the participants to understand the cumulative effect of the pandemic. Although the perceptions of some participants in the second phase were of increasing difficulty over time, comparisons between the first and second phases were not possible due to two different groups of participants. Nevertheless, despite these limitations, many participants engaged enthusiastically with the research as an opportunity to share their experiences, stories and insights. Analysis revealed not only the challenges met by an often-marginalised group, but also how these challenges arose due to threats to the fundamental dimensions of human existence we all share. As such, this analysis suggests that Byrne and Wykes’ observation in May 2020 still has resonance:

*Never has there been a greater opportunity to stop pathologizing the emotional experiences of human beings and start connecting over commonality, sharing stories and strategies to collectively work our way forward. As a global community, we are all engaging with personal recovery on some level and trying to create a new life, with meaning and hope, beyond the effects of Covid 19 (2020, p. 243).*

## Footnotes

1 P1/P2 indicates the phase of the study during which the participant was interviewed.
